# A Challenging Case of Recurrent Fever

**DOI:** 10.7759/cureus.67216

**Published:** 2024-08-19

**Authors:** Catarina Andrade, Beatriz Câmara, Ricardo Figueira, Isabel Esteves, António Jorge Cabral

**Affiliations:** 1 Pediatrics, Hospital Central do Funchal, Serviço de Saúde da Região Autónoma da Madeira, Entidade Pública Empresarial da Região Autónoma da Madeira (SESARAM, EPERAM), Funchal, PRT; 2 Rheumatology, Hospital Central do Funchal, Serviço de Saúde da Região Autónoma da Madeira, Entidade Pública Empresarial da Região Autónoma da Madeira (SESARAM, EPERAM), Funchal, PRT; 3 Pediatrics, Hospital de Santa Maria, Unidade Local de Saúde Santa Maria, Lisbon, PRT

**Keywords:** colchicine, glucocorticoids, hereditary autoinflammatory diseases/diagnosis, hereditary autoinflammatory diseases/classification, fever/diagnosis, child

## Abstract

Syndrome of undifferentiated recurrent fever (SURF) includes heterogeneous episodes of systemic inflammation without documented infection, without response to antibiotherapy, and characterized by a paucity of specific clinical or molecular criteria. Colchicine is an effective treatment with an impact on morbimortality.

We describe a case of a previously healthy one-year-old male, with consanguineous ancestry, admitted four times due to recurrent fever, associated with nonspecific symptoms and an increase of inflammatory markers in a sepsis-like pattern. No consistent infection was documented, and there was no response to broad-spectrum antibiotics. The evolution revealed corticosteroid dependency. The autoinflammatory syndrome-targeted next-generation sequencing (NGS) gene panel didn't detect relevant pathogenic variants. SURF was postulated as a diagnosis of exclusion, and the effectiveness of colchicine supports an autoinflammatory etiology.

We aimed to draw attention to recurrent fevers associated with autoinflammatory disorders due to their challenging diagnosis. Improved understanding of immune pathways and advances in genetic testing will enable greater accuracy in the approach.

## Introduction

Recurrent fevers in pediatrics have a lengthy differential diagnosis. Although acute and chronic infections are frequently considered early, they may also suggest malignancy, autoimmunity, immunodeficiency, and autoinflammatory syndromes [1]. The classic autoinflammatory disorders are defined as intermittent episodes of systemic inflammation and fever in which pathogenic inflammation arises primarily through the antigen-independent hyperactivation of immune pathways, without evidence for infection and with no role for autoreactive cells or autoantibodies [1,2].

Autoinflammatory diseases result from inborn errors in the innate immune system including hyperactivation of inflammasomes and gene mutations that are directly or indirectly involved in the regulation of the interleukin (IL-1) cytokine signaling pathway [3,4].

First recognized just over two decades ago, the autoinflammatory disease spectrum comprises the following: hereditary recurrent fevers (HRF) secondary to monogenic defects; periodic fever, aphthosis, pharyngitis, and adenitis (PFAPA), a non-hereditary multifactorial disorder; and syndrome of undifferentiated recurrent fever (SURF), a heterogeneous group of confounding conditions that are emerging [5]. Proposed empirical indications for the clinical suspicion of SURF include normal growth and development; recurrent fevers with elevated inflammatory markers (three similar episodes of fever of unknown origin in six months); monthly episodes with three to five days of duration; associated symptoms of fatigue/malaise, arthralgia/myalgia, pharyngitis, abdominal pain, and eye manifestations; asymptomatic and with normal acute-phase reactants between febrile episodes; negative criteria for PFAPA; negative genotype for HRF; and continuous colchicine/anti-IL-1 response [6,7].

The etiology underlying the recurrent fevers in SURF remains unknown. Therefore, the diagnostic approach and the most effective therapy are best addressed on an individual basis [6].

We share the case of a one-year-old child with corticoid-dependent recurrent fevers in whom SURF is hypothesized as a diagnosis of exclusion. 

## Case presentation

The patient is a one-year-old male, with normal development and growth. He is the first child of a healthy consanguineous couple (first-degree cousins), with a maternal half-sister diagnosed with celiac disease. There was no relevant known epidemiological context.

At 20 months old, he was hospitalized after a four-day history of fever (maximum temperature of 40°C) and was treated with amoxicillin 90 mg/kg/day for acute otitis media in the previous two days, without improvement. There were no relevant findings from the physical examination. The laboratory evaluation revealed anemia, neutrophilic leukocytosis, coagulopathy, and elevation of C-reactive protein (CRP) 424.79 mg/L and procalcitonin (PCT) 26.78 ng/mL (Table 1). Sepsis was presumed, and ceftriaxone IV 100 mg/kg/day was initiated, with posterior escalation on the third day due to persistent fever associated with exudative tonsillitis. Blood, urine, and throat swab cultures were negative. On the fifth day, incomplete Kawasaki syndrome (KS) was assumed, considering the nine-day history of fever, with mucositis and cervical lymphadenopathy, corroborated by analytical parameters (Table 1). Cardiac abnormalities were excluded by echocardiography. An anti-inflammatory dose of acetylsalicylic acid 30 mg/kg/day was initiated, and apyrexy was obtained after two doses of intravenous immunoglobulin (IVIG) 2 g/kg/day, 36 hours apart. On the 16th day of hospitalization, despite clinical improvement, laboratory evaluation revealed a hemoglobin decrease, thrombocytosis, coagulopathy, and an increase in inflammatory markers (Table 1). Methylprednisolone IV 1.2 mg/kg/day was begun with a favorable response. After five days, corticotherapy was switched to prednisolone, with progressive weaning after discharge. Prednisolone was suspended after four weeks with the complete normalization of laboratory parameters (Table 1).

**Table 1:** Evolution of laboratory parameters in different hospitalizations PT: prothrombin time; INR: international normalized ratio; aPTT: activated partial thromboplastin time; CRP: C-reactive protein; PCT: procalcitonin; ESR: erythrocyte sedimentation rate; ALT: alanine transaminase; AST: aspartate aminotransferase

Laboratory parameters (reference range)	First hospitalization			After discharge	Second hospitalization		Third hospitalization	Fourth hospitalization
	On admission	Fifth day	16th day	After four weeks of prednisolone	On admission	Reassessment in less than 24 hours	On admission	On admission
Leukocytes (4500-17000/μL)	25400/μL	27700/μL	6630/μL	12400/μL	22600/μL	4970/μL	38100/μL	30300/μL
Neutrophils (1500-11000/μL)	70.3%; 17800/μL	76.8%; 21300/μL	30.6%; 2000/μL	57.3%; 7100/μL	81.3%; 18400/μL	90%; 4500/μL	79.4%; 30300/μL	70%; 21200/μL
Lymphocytes (2000-14000/μL)	19.4%; 4900/μL	14.1%; 3900/μL	47.7%; 3200/μL	34.6%; 4300/μL	10.8%; 2400/μL	7.6%; 400/μL	8.1%; 3100/ μL	15%; 4500/μL
Platelets (144000-440000/μL)	249000/μL	264000/μL	451000/μL	369000/μL	154000/μL	104000/μL	245000/μL	210000/μL
Hemoglobin (10.1-12.7 g/dL)	10.8 g/dL	9 g/dL	7.1 g/dL	12.7 g/dL	12.8 g/dL	9.4 g/dL	13.7 g/dL	14.8 g/dL
PT (9.4-12.5 s)	22.4 s	25.2 s	17.3 s	13.1 s	26.4 s	33.6 s	22.7 s	19 s
INR (0.9-1.2)	1.93	2.17	1.48	1.11	2.23	2.83	1.85	1.57
aPTT (25-37 s)	29 s	30 s	31 s	23 s	29 s	30 s	28 s	31 s
Fibrinogen (200-393 mg/dL)	-	1015 mg/dL	592 mg/dL	217 mg/dL	753 mg/dL	1352 mg/dL	536 mg/dL	-
D-dimer (<255 ng/mL)	-	605 ng/mL	1177 ng/mL	<150 ng/mL	299 ng/mL	845 ng/mL	292 ng/mL	-
CRP (<6.10 mg/L)	424.79 mg/L	244 mg/L	45.7 mg/L	0.35 mg/L	368.31 mg/L	400.41 mg/L	158.01 mg/L	167 g/dL
PCT (high risk of sepsis >2 ng/mL)	26.78 ng/mL	1.98 ng/mL	0.15 ng/mL	-	38.12 ng/mL	31.91 ng/mL	10.66 ng/mL	0.27 ng/mL
ESR (<10 mm)	-	101 mm	138 mm	5 mm	25 mm	-	14 mm	-
Ferritin (30-400 ng/mL)	-	209 ng/mL	213 ng/mL	-	-	242 ng/mL	-	250 ng/mL
Albumin (35-48 g/L)	-	33 g/L	26.7 g/L	43.5 g/L	41.9 g/L	30.8 g/L	42.9 g/L	44 g/L
ALT (17-63 U/L)	-	11.4 U/L	4.7 U/L	18.3 U/L	60.3 U/L	44 U/L	18.5 U/L	12.4 U/L
AST (15-60 U/L)	-	17.3 U/L	21.8 U/L	25.9 U/L	81.9 U/L	60.1 U/L	38.1 U/L	29.8 U/L
Triglyceride (<150 mg/dL)		115 mg/dL	214 mg/dL	-	-	122 mg/dL	-	85 mg/dL

Twenty-four hours after corticotherapy was stopped, he restarted a fever associated with exudative tonsillitis and bilateral cervical lymphadenopathy. He was admitted with a new elevation of inflammatory markers (CRP 368.31 mg/L; PCT 38.12 ng/mL) and coagulopathy (Table 1), without response to broad-spectrum antibiotherapy. Despite good general condition, he remained sustainably feverish. Given the rapid analytic deterioration, with pancytopenia, hypergranulated neutrophils, ferritin in the normal range, elevation of hepatic cytolysis parameters, hypoalbuminemia, coagulopathy, hypokalemia, hypocalcemia, and hypophosphatemia (Table 1), he was transferred to the pediatric intensive care unit. Blood, urine, throat swab cultures, stool cultures, and parasite tests were negative. Lumbar puncture was not performed due to coagulopathy. Five pulses of methylprednisolone IV 20 mg/kg/day were administered, with apyrexia after the first and progressive laboratory improvement. He was maintained on prednisolone 1.5 mg/kg/day.

In the context of recurrent fever with two months of evolution, the complementary evaluation described in Table 2 was performed. By interpretation of Epstein-Barr virus (EBV) serologies, the diagnostic hypothesis of hemophagocytic lymphohistiocytosis (HLH), secondary to EBV infection, was raised in a child with probable primary immunodeficiency (male and consanguinity). Next-generation sequencing (NGS) panel for primary immunodeficiencies detected in heterozygosity two variants of unknown significance TPP2:p.(Arg225Gly) and TYK2:p.(Arg448Trp).

**Table 2:** Complementary evaluation *All documented serologies were obtained prior to the administration of immunoglobulin, except for the second evaluation of EBV serologies, as indicated in the table. HIV: human immunodeficiency virus; HSV: herpes simplex virus; CMV: cytomegalovirus; EBV: Epstein-Barr virus; VCA: viral capsid antigen; EBNA: Epstein-Barr nuclear antigens; CT: computed tomography

Diagnostic complementary tests*	Results
Ag/Ac HIV	Negative
HSV	IgM negative and IgG positive
CMV	IgM negative and IgG positive
Parvovirus	IgM negative and IgG positive
Enterovirus	IgM and IgG negative
Measles	IgM negative and IgG positive
Rubella	IgM negative and IgG positive
Bartonella henselae	IgM and IgG negative
Mycoplasma pneumoniae	IgM and IgG negative
Real-time reverse transcriptase quantitative polymerase chain reaction for SARS-CoV-2	Negative
Serologies for SARS-CoV-2	Negative
EBV: prior to immunoglobulin	Anti-EBV VCA IgM inconclusive and IgG negative
EBV: six weeks after immunoglobulin and under corticotherapy	Anti-EBV VCA IgM negative and IgG positive EBNA positive
Plasma EBV viral load	Negative
EBV viral load in blood marrow	Undetected
Anti-streptolysin O titers	Negative
Anti-DNase B titers	Negative
Quantitative immunoglobulins	Normal range
Lymphocyte subpopulations	Normal range
Complement components	Normal range
Autoantibodies	Normal range
Antiphospholipid antibodies	Normal range
Calprotectin	Normal range
Cytotoxic activity of natural killer cells	Low expression of perforin in the cytoplasm
Myelogram unveiled	Hemophagocytosis and erythroid hypoplasia phenomena
Bone marrow aspirate immunophenotyping	Inversion of CD4/CD8 T cells relation and increase of gamma/delta T cells, suggestive of a reactive process
Cervical and abdominal ultrasound	Cervical reactive lymphadenopathies and two biliary liver cysts
Cervical-thoracic-abdominal CT scan	Cervical reactive lymphadenopathies and two biliary liver cysts

After four months of weaning off prednisolone, corticotherapy was switched to deflazacort in an equivalent dose. One week after this change, he restarted a fever. On physical examination, aphthous stomatitis and pharyngitis were denoted, and the blood sample revealed a new increase in inflammatory markers (CRP 158.01 mg/L; PCT 10.66 ng/mL) (Table 1). Similar to the previous episode, he became apyrexial after the first pulse of methylprednisolone 20 mg/kg/day, and he was discharged under prednisolone. Cyclosporine oral 3 mg/kg/day was added as a corticosteroid-sparing agent during outpatient follow-up, one month after discharge.

One year after the first admission, he presented with a one day-history of fever, in association with pharyngitis and elevation of acute-phase reactants (CRP 167 mg/L; PCT 0.27 ng/mL) (Table 1). There was a clinical and analytical improvement after the adjustment of prednisolone and cyclosporine doses. EBV serologies were repeated, with negative anti-EBV VCA IgM and IgG. Therefore, previous seroconversion was considered a result of immunoglobulin administration. 

He was discharged medicated with prednisolone 0.75 mg/kg/day and cyclosporine 3 mg/kg/day. In follow-up, he had normal laboratory values between febrile episodes. He developed clinical findings suggestive of iatrogenic Cushing's syndrome due to prolonged exogenous administration of glucocorticoids, namely, moon facies, flushed appearance, hirsutism, and generalized obesity, without growth impairment.

At this point, the hypothesis of SURF was postulated as a diagnosis of exclusion. Colchicine was added, with subsequent withdrawal of cyclosporine and slow weaning from prednisolone. This regimen was completed uneventfully, and one year later, the child is medicated only with colchicine 0.25 mg/day with no record of new inflammatory exacerbations and with improvement in the adverse effects of glucocorticoids. The NGS panel for autoinflammatory syndromes, including 97 genes, didn't detect potentially pathogenic variants.

## Discussion

Autoinflammatory disorders presenting in childhood are increasingly recognized. A concomitant increase in the number of patients with undefined fever disorders has also been identified, with estimates of 60-80% of recurrent fever patients lacking a molecular or genetic diagnosis [6]. Epidemiologic data available suggests that even if PFAPA is by far the most frequent diagnosis, SURF is a more common diagnosis compared to HRF [8].

We report a male with no signs of poor weight gain, which is particularly common in children with chronic disease or immunodeficiency, but not described in SURF cases. Attending to the heterogeneous nature of SURF, including genetic, epigenetic, and environmental factors, consanguinity may be valorized as a risk factor in our patient. He had no family history of recurrent fevers; however, 35% of SURF cases have a family member with recurrent fevers of unknown origin in childhood that resolved over time [1].

Age at symptom presentation was 20 months, and the literature corroborates an early onset of fevers in SURF (median between three and 4.3 years in different references) [6,9]. We described four episodes of recurrent fever during a year, with a variable frequency of flares (periods of intermission ranged from one to six months). In contrast to PFAPA, febrile episodes tend to occur less frequently and are more difficult to predict [1].

Most cases of SURF described in the literature had a previous history of infection requiring antibiotic therapy, a characteristic uniformly absent in PFAPA syndrome. In contrast to recurrent infection cases, patients with SURF also experienced febrile episodes in which no infection was identified, and antibiotics were ineffective [1]. Indeed, in the first flare of the aforementioned case, the child was medicated for an otitis on admission. In the following episodes, the suspected triggers were the suspension or change of corticotherapy. In none of the episodes, an infectious etiology was identified, and there was always a lack of response to antibiotics. These were initiated mostly in the first admissions because of the laboratory findings suggestive of sepsis.

According to previous cohorts, inflammatory episodes did not have a characteristic pattern of symptoms, and SURF seems to be halfway on the clinical spectrum of disease severity ranging from PFAPA to HRF [8]. The most commonly reported symptoms are arthralgia, myalgia, fatigue, malaise, exudative pharyngitis, and mucocutaneous manifestations [9]. SURF patients were more likely to report gastrointestinal symptoms such as nausea, vomiting, and abdominal pain compared to PFAPA patients [6]. In the present case, exudative tonsillitis, aphthous stomatitis, pharyngitis, and bilateral cervical lymphadenopathy were the reported manifestations, while gastrointestinal symptoms were absent.

In the first episode, given the time of evolution of the fever and associated symptoms, it was initially assumed the hypothesis of incomplete KS. With increasing awareness of KS cases with incomplete clinical signs and of IVIG resistance with persistent fever after treatment, the distinction between recurrent KS and recurrent fever syndromes becomes more difficult. These similarities may suggest the activation of similar pathways, mediated by IL-1. The identification of a pattern of recurrent fevers with additional clinical signs becomes the most important diagnostic feature [10]. Retrospectively, this allowed the exclusion of KS diagnosis in our case.

During the clinical course, HLH was considered, considering that some patients have chronic intermittent presentations, with recurrent fevers of unknown origin that manifest only a subset of the classic diagnostic criteria. Additionally, he presented multiple possible instigating triggers, such as suspected EBV infection, hypothesized KS, and consanguinity, which constitute a risk for inherited immunodeficiency disorders and mutations in HLH-associated genes [11,12]. Despite the presence of fever, cytopenia, and hemophagocytosis in the bone marrow, he did not fulfill the required diagnostic criteria according to the HLH-2004 trial [13]. In parallel, the genetic test was negative for HLH-associated mutations. Presently, it was admitted that the EBV seroconversion was probably an analytical interference secondary to IVIG infusion.

The laboratory study performed, including immune evaluation, was normal. The literature emphasizes that a diagnosis cannot be established in nearly 20% of cases of highly elevated inflammatory markers, making pattern recognition of autoinflammatory features critical [14].

NGS technologies are revolutionary diagnostic tools for nonspecific immune dysregulatory phenotype, allowing the simultaneous analysis of different genes associated with a given group of inherited disorders [15]. The clinical interpretation of large panels is complicated by the detection of numerous variants of uncertain clinical significance, whose impact on the function of the gene product is unrecognized [16]. It is currently unknown whether SURF patients represent autoinflammatory gene variants of known syndromes, complex genetic autoinflammatory disorders, or independent entities [1]. Massive sequencing, therefore, represents a powerful approach to enable the definition of the molecular etiology of SURF [15].

Consistent with the variability in symptoms, patients also have varied responses to therapy [1]. Steroids are an effective strategy to control symptoms in a high proportion of patients [8]. However, attending to the important side effects of corticotherapy at high doses or frequency, daily oral colchicine was postulated as a useful alternative strategy to control symptom recurrence. Response to colchicine supports an autoinflammatory etiology, because it can attenuate the assembly of the inflammasome and thus treat familial Mediterranean fever (FMF) as well as an appreciable fraction of suspected autoinflammatory syndromes that defy molecular diagnosis [2]. According to the literature, colchicine is a low-cost, safe, and effective treatment approach for SURF, enabling the reduction of morbidity and mortality, as it can result in a significant decrease in systemic inflammation with subsequent reduction of disease activity, flare frequency, and flare duration [17]. On the other hand, cyclosporine has been historically used in monogenic autoinflammatory diseases, but with inconsistent results [18].

Currently, the median time for the follow-up of SURF cases described in the literature is too short to estimate long-term prognosis. Nevertheless, considering that a significant proportion of SURF patients exhibit both recurrent aphthous ulcers and abdominal or musculoskeletal pain, it is still possible that part of them will develop a Behçet disease on a longer follow-up [8].

## Conclusions

Our case emphasizes that chronic autoinflammatory disorders should be considered even in children with no relevant medical history and normal growth. Given the rarity of autoinflammatory disorders and the nonspecific clinical and analytical features, recurrent fevers remain a diagnostic challenge. Improved understanding of autoinflammation and advances in genetic testing may illuminate new immune pathways and provide novel diagnostic and therapeutic possibilities.
